# 

**Published:** 1893-08-26

**Authors:** 


					344 THE HOSPITAL. Aug. 26, 1893.
OUR NEW OFFICES,
428, Strand, W.C., will henceforth he the Office of this Jonrnal.
Notices of Births, Deaths, and Marriages are incited in
this colnmn at a charge of 2s. 6d. for an announcement not exceeding
four lines.
NOTICE TO CORRESPONDENTS.
All MS., Letters, Books for Review, and other matters intended for
the Editor should be addressed The Editor, The Lodge, Porchester
Square, London, "W.
The Editor cannot undertake to return rejected MS., even when
accompanied hy stamped directed envelope.
Notice to Advertisers and Subscribers.
All Advertisements, Orders for Copies of The Hospital, and Business
Communications should be addressed to The Publisher, not to the Editor,
The Hospital, 428, Strand, W.C.
The Cost of Subscription, post free, is as follows :?Per week, 2jd.;
per quarter, 2s. 6d.; per half-year, 5s.; per annum, 8s. 6d. Foreign :?
Per Annum, 10s. 8d.; payable, in all cases, in advance.
"Burdett's Hospital Annual," 1893.
Fourth year of publication. 700 pp. Boards, gilt lettering. A most
complete guide to British, Colonial, Indian, and American Hospitals,
Dispensaries, Nursing and Convalescent Institutions, and Asylums.
Price 5s. Published by The Scientific Press (Limited), 428, Strand,
W.C.
NOTICE.?The Index for Vol. XIII. (ending March, 1893)
is on sale at the Office of this Journal, price 2id.,
post free.
Scraps and Gleanincs.
The Plumpton Convalescent Home at Brighton has benefited 132
children during the past year.
It is proposed to hold a conrso of Ambulance and Health] Lectures at
Doncaster during the winter months.
It is estimated that fruit growers in the United States lose yearly
4,000,000 dollars through the insect pests.
The number of medical students at the combined universities of
Austria was 5,471, as officially stated for the Semester of 1892-3.
Much public dissatisfaction is being expressed at Barnstaple against
the sites which are proposed for the Infectious Diseases Hospital.
From Prague we learn that Dr. Wenzel Kopfstein has caused a
successful implantation of cancerous tissue in the brain of a rabbit.
A temporary hospital for small-pox cases was recently fitted up at
Moor Top, Meltham. inthe Colne Valley, at a cost of about ?200.
One hundred thousand dollars has been bequeathed to the German
Hospital at Philadelphia through the will of the late Herr A. J. Drexel.
The proposed en largement of the Essex and Colchester Hospital will
include a children's ward, a new laundry, and increased accommodation
for nurses.
The medical men of Blackpool have contributed ?100, in the name of
the Fylde Medical Society, towards the funds of the new hospital build-
ing account.
An appeal is being made for a new ward to the North-side Hospital
(Sunderland), which is required to provide additional accommodation for
women and children.
A new promenade is to be given to Londoners. The Tower Walk is,
by the Queen's command, to be thrown open to the publio every day tilt
October 1st as an experiment.
Articles of gentlemen's cast-off wearing apparel will be accepted
gratefully by the Matron of the Surrey Convalescent Homo for the use
of the male inmates of the institution.
We understand that the borough surveyor is engaged in amending th?
plans for the Infections Diseases Hospital at Mansfield to meet the re-
quirements of the Local Government Board.
Up to the present date 55,412 convalescents have benefited through
the " Home " at Dunoon. The past year has proved the most prosperous
financial epoch in the history of the institution.
Over ?316 was realised in aid of the Victoria District Nurse at Ren-
frewshire, in July, by means of an entertainment and bazaar. The
former included tableaux vivants and theatricals.
In this I year's report of the Northside Hospital, Sunderland, the sub-
scriptions from working men are shown as reaching ?549, whilst those?
from private sources have advanced 65 per cent.
The Derby and Derbyshire Convalescent Homo presented a record of a
year of useful work at the late annual meeting. There have been 190
patients received into the home during the last 12 months.
At the annual meeting of Birmingham and Midland Eye Hospital the
Committee reported the funds in a very satisfactory condition, and tho
question of extending the hospital came under consideration.
The Dispensary Sunday Festival at Pontefract is becoming a very
successful institution, and augurs well for an increased interest in th?
philanthropic work which is being accomplished by this charity.
The finances of St. Mark's Ophthalmic and Aural Hospital at Dublin,
are not in a very satisfactory condition. This is attributed mainly to
the exceptionally large amount of gratuitous relief lately given to tho
sick-poor.
At the last meeting of the Committee of the Halstead Cottage Hos-
pital it was decided to allow any medical man (who had been six months
resident in the town) to follow his patient's case after entrance into tho
hospital.
New York City has undertaken a venture of a new order, this is the
electrolysing of 120,000,000 gallons of drinking iwater, and thus purifying
it. Five horse-power dynamos are to be employed, and a 1,000 gallon
electrolysing tank.
It is gratilying for the Board of the Llandudno Cottage^ Hospital t?
be able to report that the present financial position of the institution is
in a satisfactory condition, aud that a balance to the good is shown on
last year's accounts.
A public appeal for subscriptions and donations towards the funds of
the Corbett Hospital at Stourbridge resulted in donations to the extent of
?2,248, including ?1,000 from Mr. W. O. Foster, and the annual subscrip-
tions reached ?367.
At the annual meeting of the Sarah Nicol Memorial Cottage Hospital
a favourite report for the past year was read. Financially the institu-
tion is in a flourishing condition, and two isolation wards are shortly to
be added to the existing buildings.
A suitable site has been selected for the Pontypridd Cottage Hos-
pital. The various surrounding collieries have contributed towards the
necessary funds. The hospital will have ten representatives of the
working classes on the Committee.
The corner-stone of the Blackpool Infirmary was recently laid. Iq.
has been decided to proceed at present only with that portion of tho
hospital which shall be appropriated for accident cases; the remainder
of the wards are to be built when funds will allow.
The special appeal for contributions towards the expenses of the-
recent improvements within the Royal South Hampshire Infirmary has-
failed to enlist a larger sum than ?1,156; yet this institution extends its
sphere of usefulness over a population of 100,000 persons.
New York is iyet to boast of another hospital, to be called " The
Grace," which institution will consist of three supplementary buildings,
called The House of Simeon, for old men, The House of Anna, for
old women, and The House of the Holy Child, for children.
A large National and Floral Fete was held at the beginning of the
month in Feltes College grounds, Edinburgh, in aid of the Maternal
nad Simpson Memorial Hospital. There was a caW chantant and a
gipsy encampment, as well as many other attractions calculated to
ensure tho financial success of the entertainment.
FIRST IMPRESSIONS OF BOOKS RECEIVED.
[It is requested that all books intended to be noticed in this column may
bo sent to the Editor, at the Office, 428, Strand, W.C., not later than
Tuesday evening in each week.]
A Text-book of the Theory and Practice of Medicine.
By American Teachers. Edited by William Pepper,
M.D., LL.D., Provost and Professor of the Theory and
Practice of Medicine and of Clinical Medicine in the
University of Pennsylvania. In two volumes. Illus-
trated. Vol. I. Philadelphia, W. B. Saunders, 1893.
This is an ambitious attempt to provide a text-book which
shall represent the views of some of the most experienced and
successful of the teachers of medicine in America. Dr.
Pepper, who edits it, has secured the co-operation of twelve
other teachers, and among them several of the best-known
American physicians. The first volume, which may be had
bound up in two parts, consists of some 900 large pages, so
that the entire work is on such a scale that the subjects can
be dealt with adequately, if not exhaustively. The type is
very good, the illustrations, which are few in this volume,
are good, and are mainly devoted to the delineation of micro-
organisms. The opening chapter is from the well-known pen
of Dr. J. S. Billings, who writes on hygiene, a subject on
which he is specially qualified to speak. This article occupies
some forty-four pages, and deals with many branches of the
subject, some of them quite cursorily. Dr. Billings has spoilt
Books Receiyed.
Pamphlets.?The World of Forgotten Children, Excelsior, The
Table, Religious Review of Reviews, The Christian World, British and
Colonial Druggist, The Therapist, Review of Reviews, The Medical
Chronicle.
Aug. 26, 1893. THE HOSPITAL. xi
Medical Vacancies and Appointments.
APPOINTMENTS.
r>?;' ^ams> M.D.St.And . M.R.C.S.Eng., reappointed Medical
Officer for the Barnes Sanitary District of the Richmond Union.
? Arnold, M.R.C.S.Eng., appointed Medical Officer for the
Oreat Miiton and Little Milton Districts of the Thame Union.
J*" Blacker, M.B., B.S.Lond., F.R.C.S., appointed Assistant
Obstetric Physician to University College Hospital. E. M.
?Urockbank, M.D.Vict., appointed Resident Medical Officer at
a Infirmary, Manchester. Mr. A. E. Buller, appointed
Assistant Medical Officer to the Infirmary of the Parish of St.
JLancras. K. J. Courtenay, L.R.C.P., L.R.C.S.Edin., appointed
^ledical Officer for the Wareham No. 1 and Morden Districts of
Wareham and Purbeck Union. P. V. Dodd, M.A.,
^o= ng., L.R.C.P.Lond., reappointed Assistant Medical
Officer of Health for Folkestone. Dr. Drabble, appointed
?Medical Officer for the Walton District of the Chertsey Union.
^etcher, L.R.C.P., L.R.C.S.Edin., appointed Medical
Officer for the Uttoxeter and Leigh Districts and the Workhouse
\r ^tt?xeter Union. J. S. Fowler, M.B., C.M.Edin.,
^?R.C.S.Eng., apDointed Resident Medical Officer to the Edin-
Mt?*1 Children's Hospital. J. H. Freer, L.R.C.P.Lond.,
^?R.C.S.Eng., reappointed Medical Officer of Health for
Rugeley. F. W. Fullerton, M.B.. B.S.Durh., M.R.C.S.,
?fj.R.C.P.Lond., appointed Junior Assistant House Surgeon to
t 6 Hull Royal Infirmary. A. W. German, M.R.C.S., L.R.C.P.
^?nd., appointed Assistant Medical Officer for the Mill Road
infirmary of the West Derby Union. B. C, Gowing, M.R.C.S.
^ng_., L.S.A., reappointed Medical Officer of Health for the
^enistone Urban Sanitary District. C. A. Griffiths, F.R.C.S.
L.R.C.P.Lond., appointed House Surgeon to the Swansea
general and Eye Hospital. T. H. Haydon, M.B., B.C.Camb.,
^?R.C.P.Lond., M.R.C.S.Eng., appointed Medical Officer of
Wealth for the Marlborough Urban Sanitary District of the Marl-
borough Union. C. W. Hemming, L.R.C.P., L.R.C.S.Edin.,
appointed Medical Officer of Health for the Glvncorrwg District
Local Board. H. D. Hey, M.R.C.S., L.R.C.P'Lond., appointed
^ledical Officer for theBuckland Sanitary District of theFarring-
oon Union. C. H. Higgins, M.D.St.And., M.R.C.P.Lond.,
" -R-C.S.Eng., appointed Medical Officer of Health for the Cleck-
heaton Urban Sanitary District of the North Bierly Union. J.
L. Hobbes, L.R.C.P., L.M.Edin., M.R.C.S.Eng., appointed
Medical Officer for the Belbroughton District of the Bromsgrove
Union. J. W. Hodgson, M.D.Aberd., appointed Medical Inspec-
tor of Seamen for the Port and District of Exmouth. F. D.
Holmes, B.A.R.U.I., L.R.C.P.Edin., appointed Medical Officer
for the Duffield District of the Belper Union. J. A. Hutchinson,
M.D.Durh., M.R.C.S.Eng., appointed Medical Officer for the
Northallerton District and the Workhouse of the Northallerton
Union. A. Jamison, M.A., M.B., B.Ch., B.A.O., as House-
Surgeon to the Bristol Hospital for Sick Children and Women.
H. Jones, L.S.A., D.P.H.Camb., appointed Medical Officer of
Health for the Borough of Crewe. B. Keane, L.R.C.P.,
L.R.C.S.Edin., L.F.P.S.Glasg., appointed Medical Officer for
the Western District of the Hamlet of Mile End Old Town. H.
W. Kendall, M.R.C.S., L.R.C.P.Lond., appointed House Sur-
geon to the Great Northern Central Hospital, Holloway Road,
N. J. Lane, L.R.C.P., L.R.C.S.Edin., appointed pro tern.
Medical Officer for the Timoleague Dispensary District, Cork. Mr.
J. D. Mackay, appointed Medical Officer for the Knaresborough
District of the Knaresborough Union. A. A. Mackeith, M.B.,
C.M.Glasg., appointed Medical Inspector of Seamen for the Port
and District of Exeter. N. S. Manning, L.R.C.P., F.R.C.S.Irel.,
appointed Medical Officer for the Eighth Sanitary District of the
Barnstaple Union. A. G. Minshall, M.R.C.S., L.R.C.P.Lond.,
appointed Assistant Medical Officer to the Infirmary of the Pariah
of St. Pancras. H. W. Morley, M.R.C.S., L.R.C.P., appointed
Assistant House Surgeon to the Royal Portsmouth, Portsea, and
Gosport Hospital. G. C. Peachey, L.R.C.P., L.R.C.S.Edin.,
L.F.P.S.Glasg., appointed Medical Officer of Health for the
Wantage Rural Sanitary District. J. D. Roberts, M.R.C.S.Eng.,
L.S.A.Lond., appointed Medical Officer to the Ealing Cottage
Hospital and Provident Dispensary. B. M. H. Rogers, M.B.,
B.Ch., M.R.C.S., L.R.C.P., as Physician to the Bristol Hospital
for Sick Children and Women. T. H. Smith, L.R.C.P.,
L.R.C.S.Edin., reappointed Medical Officer of Health for the
Reddish Urban Sanitary District. Dr. R. Steele, appointed
Medical Officer for the Church Eaton District of the Cannock
Union. P. R. Stevens, L.R.C.P.Lond., M.R.C.S.Eng., appointed
xii THE HOSPITAL. Aug. 26, 1893.
Medical Officer of Health for the Braintree Urban Sanitary Dis-
trict. Mr. J. H. Wallis, appointed Medical Officer for the Igburgh
District of the Swaffham TJnion. T. Webster, M.R.C.S.Eng.,
L.S.A., appointed Medical Officer for the Bewdley District of the
Kidderminster Union. C. Wintle, L.R.C.S., M.R.C.S., as
Surgical Registrar and Out-Patient Assistant to the Bristol
Hospital for Sick Children and Women. G. T. Woods,
L.R.C.P., L.R.C.S.Edin., appointed Medical Officer of Health
for the Ripley Sanitary District of the Knaresborough Union.
VACANCIES.
The following vacancies are announced this week, the amoant of
salary in each case being given after the name of the institution :
Resident medical Officers.
Bridgnorth and South Shropshire Infirmary. House Surgeon, doubly
qualified. Salary for the first year ?80 per annum, with board and
lodging. Applications to the Honorary: Secretary, Oldbury Rectory,
Bridgnorth, by August 26th.
Brighton, Hove, and Preston Dispensary, Board Room, Queen's Road,
Brighton. House Surgeon, unmarried, doubly qualified. Salary
?140 per annum, with furnished apartments, coal, gas, and
attendance. Applications to C. Somers Clarke, Honorary Secretary,
by September 1st.
Denbighshire Infirmary, Denbigh. House Surgeon ; must be conversant
with the Welsh language. Salary ?85 per annum, with board, resi-
dence, and washing. At the end of a year's engagement, if satisfac-
tory, a gratuity of ?15 is given by the committee. Applications to
W. Vaughan Jones, Secretary.
General Hospital, Birmingham. Houso Physician. Salary ?70 per
annum, with residence, board, and washing. Applications to Howard
J. Collins, House Governor, by September 2nd.
London Temperance Hospital, Hampstead Road, N.W. House Surgeon
and Junior Honse Surgeon, doubly qualified. Residenoe, board, and
washing provided. Appointments for six months. The House
Surgeon will receive a salary at the rate of 50 guineas per annum,
and the Junior House Surgeon will receive an honorarium of 5
guineas on satisfactory completion of term of appointment. Appli-
cations to E. Wilson Taylor, Secretary, by August 31st.
St. Peter's Hospital for Stone and Urinary Diseases, Henrietta Street,
Covent Garden. House Surgeon ; must be M.R.O.S.'; appointment
for six months. Honorarium 25 guineas, with board, lodging, and
washing. Applications to the Secretary by September 11th.
Sheffield General Infirmary. Assistant House Surgeon, doubly qualified.
Salary ?80 per annum, with board, lodging, and washing. Applica-
tions to the Medical Staff of the Sheffield General Infirmary, to the
oare of the Secretary, by August 26th.
Stafford General Infirmary, Stafford. Assistant House Surgeon. Board
and residence provided. Applications to the Secretary iby Septem-
ber 9th.
Teignmouth, Dawlish, and Newton Infirmary, Dispensary, and Con-
valescent Home. House iSurgeon, doubly qualified. Salary ?60
per annum, with board, lodging, and washing. Applications to the
Chairman of Committee by August 28th.
Weston-super-Mare Hospital land Dispensary. Medical Officer for the
Provident Dispensary attached to the Hospital ; doubly qualified.
Salary ?60 per annum, with board, lodging, and washing. Applica-
tions to the Honorary Secretary by August SOth.
Wolverhampton and Staffordshire General Hospital, Wolverhampton.
House Surgeon. Salary ?100 per annum, with board, lodging, and
washing. Appointment for one year, but eligible for re-election.
Applications endorsed " Application for House Surgeon " to Edwin
A. White, House Governor and Secretary, by August 28th.
Non-Resident Medical Officers.
Anderson's College Medical School, Glasgow. Chair of Materia Medica
and the Chair of Physics. Applications to John Kidstone, Secre-
tary, 50, West Regent Street, Glasgow, bv August Slst.
Borough of Chesterfield. Medical Officer of Health for the Urban Sani-
tary District. Salary ?200 per annum. Applications endorsed
"?Medical Officer " to John Middleton, Town Clerk, by Septem-
ber 4th.
National Dental Hospital, Great Portland Street, W. Assistant
Dental Surgeon. Applications to E. Almack, Secretary, by August
22nd.
Sheffield School of Medicine. Tutor to take charge of dissecting room
and to hold classes in anatomy and physiology. Salary ?125 per
annum. Applications to the Secretary by August 31st.
EXAMINATION IIT EDINBURGH FOB THE TRIPLE
QUALIFICATION.
PAPERS SET IN THE SECOND EXAMINATION
Questions in Advanced Anatomy (all of which are to be answered).
1. Describe step by step the dissection necessary to expose the Ingui-
nal Canal. State the exact position and limits of the Canal, and the
relative position of its contents.
2. Describe the anastomoses with other Arteries formed by the
branches of (1) the Thyroid Axis, (2) the Superior Intercostal Artery,
(3) the Profunda Femoris.
3. Describe the position, shape, blood-vessels, and relations to other
viscera of the Pancreas. How would you expose its duct ?
4. Describe the structure and connections of the Iris, and describe its
vascular and nervous supply.

				

